# Exploring the influencing effect of tertiary teachers’ perceived organizational support and teacher self-efficacy on their job satisfaction: the mediating role of teacher work engagement

**DOI:** 10.3389/fpsyg.2025.1609824

**Published:** 2025-09-25

**Authors:** Mei Chen

**Affiliations:** Guangxi University of Finance and Economics, Nanning, China

**Keywords:** perceived organizational support, teacher self-efficacy, teacher work engagement, teacher job satisfaction, tertiary teachers

## Abstract

Teacher job satisfaction is a central concern within the teaching profession, garnering significant global attention. This research investigates the relationships among perceived organizational support, teacher self-efficacy, work engagement, and job satisfaction among tertiary teachers in Western China. Using a survey methodology, data were collected from 450 teachers at public universities. The findings revealed significant positive correlations among all variables. Crucially, work engagement was found to partially mediate the positive relationship between perceived organizational support and job satisfaction, as well as between teacher self-efficacy and job satisfaction. These results contribute to existing theory by highlighting the pivotal role of work engagement and offer practical insights for enhancing teacher satisfaction. Recommendations are provided for policymakers, administrators, and teacher education programs. The study concludes by suggesting avenues for future research, such as incorporating additional cognitive and non-cognitive factors to deepen the understanding of job satisfaction determinants.

## Introduction

Teacher job satisfaction has emerged as a central concern in the teaching profession, attracting increasing attention from scholars and practitioners globally (Han et al., 2020; Wang et al., 2024a; Zhang et al., 2023a). While the COVID-19 pandemic exacerbated this issue through abrupt shifts to online teaching and increased workloads (Hashim et al., 2020), Recent UNESCO (2023) reports indicate that teacher attrition rates in underdeveloped regions have reached 23% annually—triple the global average—primarily driven by compounding systemic inequities. The decline in satisfaction is particularly acute in these regions, where chronic pressures—such as digital infrastructure gaps (only 12% smart classroom coverage) and misaligned incentive systems (80% compensation tied to research output)—intersect with pandemic-related disruptions. This crisis directly threatens UN Sustainable Development Goal 4’s quality education targets (Teng and Zhang, 2024), necessitating urgent context-specific interventions. For instance, in Western China, where this study is situated, tertiary institutions grapple with insufficient funding for digital infrastructure (only 12% of classrooms are equipped with smart technologies), limited access to professional development (67% of teachers report no training in blended pedagogy), and misaligned salary-reward systems where 80% of compensation is tied to research output rather than teaching quality (Wang and Chen, 2024b; Kumar and Singh, 2023a; Zhang and Li, 2023). These structural barriers amplify role conflicts between teaching and research responsibilities, creating a unique ecosystem of stressors that distinguish underdeveloped contexts from more resourced environments.

Existing research has consistently highlighted the roles of perceived organizational support (POS), work engagement, and teacher self-efficacy in shaping job satisfaction. Supportive institutional environments have been shown to foster commitment and reduce role strain (Bibi et al., 2019; Maan et al., 2020), while engaged teachers report greater fulfillment and resilience (Côté et al., 2021). However, these findings predominantly derive from studies in developed regions, where stable resources and institutional frameworks buffer workplace stressors. In contrast, underdeveloped educational systems face compounded challenges: POS may be constrained by budgetary limitations (e.g., 60% of universities in Western China operate below national funding benchmarks), self-efficacy may erode due to inadequate training opportunities (only 28% of teachers receive annual skill upgrades), and work engagement may fluctuate with unpredictable policy shifts (Li and Shao, 2018; Ji and Zhao, 2021; Guo et al., 2023). For example, teachers in low-resource settings often navigate classrooms without updated materials or technological support, undermining both their self-efficacy and organizational trust (Zhang and Li, 2023). Yet, few studies have systematically integrated these variables to explore their synergistic effects in such contexts, leaving a critical gap in understanding how systemic inequities modulate psychological and organizational dynamics.

This gap is further underscored by the Job Demands-Resources (JD-R) model (Bakker and Demerouti, 2017), which posits that job resources (e.g., POS) and personal resources (e.g., self-efficacy) buffer demands to enhance engagement. While this framework has illuminated workplace well-being in corporate settings, its application to underdeveloped educational systems remains contentious. Critics argue that the JD-R model underestimates the structural inertia of resource-poor environments, where even high self-efficacy may falter against systemic barriers like outdated curricula or bureaucratic inefficiencies (Wang et al., 2024b; Lauermann and Berger, 2023a; Xu et al., 2023). For instance, a teacher’s belief in their instructional capabilities (self-efficacy) may not translate to job satisfaction if classroom resources are chronically lacking—a scenario rarely addressed in existing JD-R research. Similarly, the mediating role of work engagement between POS and satisfaction, observed in broader organizational studies (Côté et al., 2021), remains undertested in settings where institutional support is fragmented or inconsistent.

Recent studies have begun addressing these limitations. For example, Kumar and Singh (2023a) demonstrated that in Indian higher education, POS mitigates burnout only when coupled with policy reforms addressing salary disparities—a finding challenging the JD-R assumption that resources universally buffer demands. Similarly, Zhang et al. (2023b) found that in rural Chinese schools, self-efficacy predicts job satisfaction only when mediated by community support networks, highlighting the need to integrate macro-level factors into psychological models. These insights align with calls for context-sensitive extensions ofthe JD-R framework (Lauermann and Berger, 2023a; Fackler et al., 2022), yet empirical integration remains sparse.

This study proposes an integrated model to address these gaps, examining how work engagement mediates the relationships between POS, self-efficacy, and job satisfaction specifically in underdeveloped regions. By focusing on tertiary teachers in Western China—a region characterized by rapid educational expansion amid persistent resource constraints—the research aims to uncover context-specific mechanisms that drive teacher well-being. The findings will not only refine theoretical frameworks like the JD-R model but also inform targeted interventions to enhance retention and sustainable development in resource-scarce environments.

## Literature review

### Perceived organizational support

Perceived organizational support (POS) is a critical factor influencing employee well-being and organizational outcomes. Initially defined by Eisenberger et al. (1986), POS refers to employees’ beliefs about how much their organization values their contributions and cares for their well-being. POS is positively associated with organizational commitment, job satisfaction, and work engagement (Ridwan et al., 2020; Xu and Yang, 2018), while also enhancing self-efficacy and work engagement (Han et al., 2018; Al-Hamdan and Bani Issa, 2021). Conversely, low POS can lead to job stress, role conflicts, absenteeism, and turnover (Rineer et al., 2017; Mehrad et al., 2020). For higher education teachers, POS significantly reduces job stress, burnout, and turnover intention (Kumar and Singh, 2023a; Zhang et al., 2023a; Wang and Chen, 2024), while fostering job satisfaction and performance through fair treatment and recognition (Zhang and Li, 2023). Crucially, POS not only directly boosts job satisfaction but also indirectly shapes outcomes by interacting with personal resources like teacher self-efficacy—a concept explored next.

### Teacher self-efficacy

Building on organizational resources like POS, teacher self-efficacy—a key personal resource—further elucidates the psychological pathways to educator well-being. Self-efficacy, as conceptualized by Bandura (1997), represents an individual’s belief in their capacity to execute tasks and achieve desired outcomes, serving as a critical determinant of cognition, emotion, motivation, and decision-making processes. Within the educational domain, teacher self-efficacy specifically denotes educators’ confidence in their ability to design and implement effective instructional practices that enhance student learning outcomes (Perera et al., 2019). This construct is typically operationalized through three dimensions: classroom management, student engagement, and instructional strategies (Tschannen-Moran and Hoy, 2001).

In recent years, the education sector has undergone significant disruptions, leading to transformative changes in the quality of learning. Amidst this shift, teacher self-efficacy has emerged as a prominent and increasingly researched topic in higher education Fackler (Lauermann and Berger, 2023a; Malmberg and Sammons, 2022) reflecting its critical role in shaping educational outcomes and teaching practices. Empirical evidence underscores the significant influence of teacher self-efficacy on increased student engagement and improved academic performance (Burić and Kim, 2020; Lauermann and Berger, 2021), goal-setting, and professional productivity (Karakaş and Erten, 2021; Fackler et al., 2021), with robust associations observed between self-efficacy and job satisfaction, work engagement, and resilience (Liu et al., 2020; Granziera and Perera, 2023). Importantly, high self-efficacy not only sustains job satisfaction but also amplifies work engagement, creating a synergistic effect with organizational support—a theme expanded in the discussion of work engagement.

### Teacher work engagement

As a mediator between resources and satisfaction, teacher work engagement integrates the influences of both POS and self-efficacy, forming a core component of the JD-R model. Teacher work engagement, defined as the holistic investment of cognitive, emotional, and social resources into pedagogical roles (Schaufeli and Bakker, 2004), has been predominantly framed within the Job Demands-Resources (JD-R) model (Bakker and Demerouti, 2017). While the JD-R model provides a robust framework for understanding engagement dynamics—by categorizing workplace factors into job demands (e.g., workload, administrative burdens) and job resources (e.g., autonomy, supervisory support)—its application in educational contexts remains contentious. Critics argue that the model oversimplifies the unique challenges faced by teachers, particularly in underfunded systems where structural constraints (e.g., outdated curricula, bureaucratic inefficiencies) amplify demands while limiting resource accessibility (Wang et al., 2024b; Lauermann and Berger, 2023b). For instance, while professional development opportunities may buffer job demands in corporate settings (Bakker and Demerouti, 2017), their efficacy in resource-scarce educational environments remains debated. A recent study in Western China found that only 28% of teachers could access training programs due to budgetary constraints, rendering such “resources” largely symbolic (Guo et al., 2023). This structural inertia challenges the JD-R assumption that resources universally mitigate demands, highlighting the need for context-sensitive theoretical extensions. This engagement directly channels the benefits of resources like POS and self-efficacy into job satisfaction, highlighting its pivotal mediating role—a connection detailed in subsequent sections on job satisfaction and mediation effects.

Empirical research on the mediating role of work engagement further reveals critical inconsistencies. While some studies report strong mediation effects between organizational support and job satisfaction (Ji and Zhao, 2021), others find weak or context-dependent relationships (Dewi et al., 2021). For example, Ji and Zhao (2021) demonstrated that work engagement fully mediated the POS-satisfaction link among Tibetan primary school teachers, attributing this to collectivist cultural norms that enhance resource-sharing. In contrast, Dewi et al. (2021) observed a nonsignificant mediation effect in Indonesian vocational schools, where chronic underfunding eroded both engagement and satisfaction despite high organizational support. These discrepancies may stem from methodological and contextual differences: cross-sectional designs (e.g., Dewi et al., 2021) often conflate causation with correlation, while cultural biases in sampling—most evidence derives from Western or developed regions—neglect systemic inequities in underresourced systems (Kumar and Singh, 2023a). Additionally, the JD-R model’s focus on individual-level factors overlooks macro-level drivers such as policy reforms. In collectivist societies like China, societal expectations to prioritize institutional goals over personal well-being may paradoxically suppress engagement even when resources are available (Zhang and Li, 2023).

These limitations underscore the need for integrative frameworks that account for both psychological and structural determinants of engagement. Recent studies propose augmenting the JD-R model with systemic resource thresholds—minimum levels of institutional support required for psychological resources to translate into engagement (Xu et al., 2023). For instance, Wang et al. (2024b) found that in Western Chinese universities, teacher self-efficacy predicted engagement only when coupled with baseline classroom technologies (e.g., smart boards), suggesting that personal and job resources operate synergistically rather than independently. Such insights challenge the JD-R model’s universal applicability and call for context-driven refinements to address the unique challenges of underdeveloped educational systems.

### Job satisfaction

Ultimately, job satisfaction serves as the outcome variable, directly influenced by work engagement and indirectly by antecedent resources like POS and self-efficacy. Job satisfaction, a multidimensional construct, has been defined and interpreted in various ways across the literature. Locke (1976) conceptualizes it as a positive emotional state resulting from an individual’s evaluation of their job, while Spector (1997) views it as an attitudinal measure reflecting one’s feelings toward their work. More recently, Alonderiene and Majauskaite (2016) emphasize job satisfaction as a blend of emotions and attitudes toward the work environment, and Klettner et al. (2018) describe it as the alignment between employees’ expectations and their actual job experiences. From a cognitive perspective, job satisfaction is often framed as an analytical assessment, focusing on the discrepancy between what employees receive and what they expect from their roles (Oshagbemi, 2003). Weiss (2002) and Rose (2001) further highlight its dual nature, distinguishing between intrinsic satisfaction (emotional enjoyment derived from the job itself) and extrinsic satisfaction factors such as compensation and working condition. Psychological factors, particularly work engagement and self-efficacy, have been shown to positively influence job satisfaction (Ma et al., 2018; Sokmen and Kilic, 2019), reinforcing how resource-driven pathways culminate in this key outcome—a dynamic further examined in the mediation analysis.

Individual factors, such as personal values, expectancy beliefs, and the alignment between personal and organizational goals, also contribute significantly to job satisfaction (Affum-Osei, 2019; Wang et al., 2024a). Psychological factors, particularly work engagement and self-efficacy, have been shown to positively influence job satisfaction (Stefanidis and Strogilos, 2019; Sokmen and Kilic, 2019). Teachers who possess strong self-efficacy—believing in their ability to manage challenges and succeed in their roles—tend to report higher levels of job satisfaction (Burić and Moe, 2020; Demir, 2020). Recent studies have further highlighted the mediating role of work engagement in the relationship between self-efficacy and job satisfaction, emphasizing the importance of fostering both psychological and organizational resources to enhance teacher well-being (Granziera and Perera, 2023; Zhang et al., 2023c).

### The mediating efect of teacher work engagement

Synthesizing the preceding concepts, teacher work engagement emerges as the linchpin that explains how POS and self-efficacy jointly foster job satisfaction—core to this study’s theoretical framework. Teacher work engagement has gained increasing attention as a critical mediator in the relationships between teacher self-efficacy, perceived organizational support (POS), and job satisfaction. Recent studies have highlighted the role of work engagement in translating individual and organizational resources into positive job outcomes. For instance, Perera et al. (2019) demonstrated that work engagement partially mediates the relationship between teacher self-efficacy and job satisfaction, suggesting that teachers with higher self-efficacy are more likely to be engaged in their work, thereby experiencing greater job satisfaction. Similarly, Ji and Zhao (2021) found that work engagement serves as a significant mediator between POS and job satisfaction among Tibetan primary school teachers, emphasizing the importance of organizational support in fostering engagement, which in turn enhances satisfaction.

The Job Demands-Resources (JD-R) model provides a theoretical foundation for understanding these mediating mechanisms. According to Bakker and Demerouti (2017), job resources such as POS and personal resources like self-efficacy enhance work engagement by reducing job demands and increasing motivation. Li and Shao (2018) further supported this by showing that work engagement mediates the relationship between positive environmental factors (e.g., professional development opportunities) and occupational well-being. More recently, Zhang et al. (2023a) found that work engagement not only mediates the direct effects of self-efficacy and POS on job satisfaction but also amplifies these effects in underdeveloped educational contexts, where systemic challenges often exacerbate workplace stressors.

Despite these insights, inconsistencies remain in the literature. For example, Dewi et al. (2021) reported a positive but statistically insignificant relationship between work engagement and job satisfaction, suggesting that contextual factors such as cultural differences and resource availability may influence these dynamics. Additionally, Wang et al. (2024b) highlighted the need for longitudinal studies to better understand the causal pathways linking self-efficacy, POS, work engagement, and job satisfaction. Overall, the evidence underscores the pivotal role of work engagement as a mediator, bridging individual and organizational factors to enhance teacher well-being and job satisfaction.

### Hypothesis development

**Figure 1:**
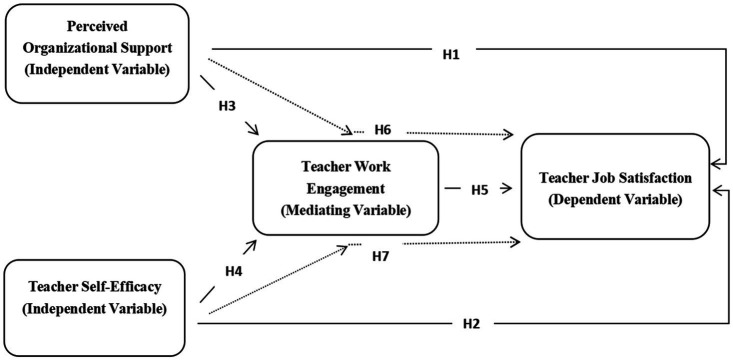
Hypothesized model of perceived organizational support, teacher self-efficacy, teacher work engagement and teacher job satisfaction.

Based on the emerging insights from recent research on perceived organitional support, teacher self-efficacy, teacher work engagement and teacher job satisfaction, the following three research hypotheses were formulated for the present study, and the hypothesized model is illustrated in Figure 1.

Hypothesis 1: There is a significant relationship between perceived organizational support and teacher job satisfaction.

Hypothesis 2: There is a significant relationship between teacher self-efficacy and teacher job satisfaction.

Hypothesis 3: There is a significant relationship between perceived organizational support and teacher work engagement.

Hypothesis 4: There is a significant relationship between teacher self-efficacy and teacher work engagement.

Hypothesis 5: There is a significant relationship between teacher work engagement and teacher job satisfaction.

Hypothesis 6: Teacher work engagement plays a mediating role between perceived organizational support and teacher job satisfaction.

Hypothesis 7: Teacher work engagement plays a mediating role between the teacher self-efficacy and teacher job satisfaction.

## Methodology

### Participants

This study involved tertiary teachers who were randomly selected from universities in Western China. A total of 481 questionnaires were distributed and collected in person during class breaks. After removing invalid responses, 450 valid questionnaires were retained, resulting in a validity rate of 93.56%. Out of 450 teachers surveyed, 134 (29.8%) were male and 316 (70.2%) were female, with the majority (55. 1%) aged 30–40. Most held master’s degrees, and 63.3% specialized in humanities. In terms of professional ranking, 51.8% were at the intermediate level, while 42.9% had 11–20 years of teaching experience. Additionally, 58.9% reported spending 31–40 h on teaching preparation per week. Prior to the survey, teachers were informed about the voluntary nature of their participation, and the process was conducted anonymously. Informed consent was obtained from all participants, who were encouraged to actively take part in the questionnaire.

### Instruments

#### Perceived organizational support

The measurement of perceived organizational support (POS) was adapted from the 22- item scale developed by Ling et al. (2006), originally validated in multinational corporate contexts. To align with the unique challenges faced by tertiary teachers in underdeveloped regions, a four-stage adaptation process was implemented. First, the three core dimensions of the original scale—work support, value recognition, and concern for employee well-being—were retained but recontextualized for academic settings. For example, the item “My company supports my professional development” was revised to “My university provides resources to balance teaching and research responsibilities” to reflect the dual role expectations in higher education. Second, a panel of five educational psychologists reviewed the scale to eliminate culturally incongruent items (e.g., “My organization cares about my family needs”), as institutional support in Chinese universities primarily focuses on professional rather than familial domains. Third, pilot testing with 50 tertiary teachers from Western China identified three items with factor loadings below 0.60 (e.g., “Leadership prioritizes my salary growth”), which were subsequently removed to enhance psychometric robustness. Finally, the refined 19-item scale was translated into Mandarin using back-translation (Brislin, 1986) and validated through confirmatory factor analysis (CFA). The final scale comprised: Work Support (9 items, e.g., “My department values my teaching philosophy”), Value Recognition (6 items, e.g., “Colleagues acknowledge my pedagogical innovations”), and Concern for Well- being (7 items, e.g., “Administrators adjust workloads during health crises”).

Reliability tests demonstrated strong internal consistency (Cronbach’s *α* = 0.939 overall; subscales: 0.914, 0.880, 0.905). CFA results confirmed structural validity (χ^2^/df = 2.423, RMSEA = 0.056, CFI = 0.949), with factor loadings ranging from 0.662 to 0.826. Convergent validity was supported by composite reliability (C*R* = 0.831) and average variance extracted (AVE = 0.622), exceeding established thresholds (Fornell and Larcker, 1981). This adaptation process ensured theoretical fidelity to Ling et al.’s (2006) framework while addressing the institutional realities of underdeveloped educational systems.

### Teacher self-efficacy

Building on Tschannen-Moran and Hoy's (2001) scale, this study adapted and developed a questionnaire to measure teacher self-efficacy. The scale utilized a 9-point Likert format, where 1 represents “None at all” and 9 represents “A great deal.” The scale encompasses three dimensions: teaching strategy (7 items, e.g., To what extent can you use a variety of assessment strategies?), classroom management (7 items, e.g., How much can you do to calm a student who is disruptive or noisy?), and student engagement (7 items, e.g., How much can you do to motivate students who show low interest in schoolwork?). The overall Cronbach’s alpha for the scale was 0.937, with the subscales for teaching strategy, classroom management, and student engagement yielding values of 0.909, 0.901, and 0.890, respectively. These results indicate that the scale has excellent reliability.

In the confirmatory factor analysis, the fit indices were as follows: χ^2^/df = 2.421, RMSEA = 0.056, SRM*R* = 0.039, NFI = 0.922, CFI = 0.952, TLI = 0.946, and IFI = 0.953. These results indicate that the confirmatory factor analysis model for teacher self-efficacy demonstrates a good fit. Additionaly, the factor loadings for teacher self-efficacy ranged between 0.824 and 0.747, with a construct reliability (CR) of 0.827 and an average variance extracted (AVE) of 0.614, indicating that the questionnaire demonstrates good convergent validity.

### Teacher work engagement

In this study, the Utrecht Work Engagement Scale (UWES), one of the most widely used scales for measuring work engagement, was adapted and further developed. Originally created by Schaufeli et al. (2002), the UWES includes 17 items that assess work engagement using a five-point Likert scale, with responses ranging from 1 (strongly disagree) to 5 (strongly agree). The scale is divided into three dimensions: vigor (6 items, e.g., “I can continue working for very long periods at a time”), dedication (5 items, e.g., “My job inspires me”), and absorption (6 items, e.g., “I am immersed in my work”). Internal consistency tests revealed Cronbach’s *α* coefficients of 0.800, 0.880, and 0.877 for the vigor, dedication, and absorption subscales, respectively. The overall Cronbach’s α for the entire scale was 0.925, demonstrating excellent reliability of the questionnaire.

The results of the confirmatory factor analysis revealed the following model fit indices: χ^2^/df = 2.469, RMSEA = 0.057, SRM*R* = 0.037, NFI = 0.933, CFI = 0.959, TLI = 0.951, and IFI = 0.959. All these indices met or exceeded the recommended thresholds, demonstrating that the confirmatory factor analysis model exhibits an excellent fit to the data. Additionally, the factor loadings ranged from 0.877 to 0.842, with a Composite Reliability (CR) value of 0.894 and an Average Variance Extracted (AVE) value of 0.737,indicating strong convergent validity and composite reliability.

### Job satisfaction

By adopting the Minnesota Satisfaction Questionnaire (MSQ), this study explored a 17 items scale for teacher job satisfaction, ranging from 1 (dissatisfied) to 5 (very satisfied). there are two sub-scales, namely intrinsic satisfaction (9 items, e.g., Being able to keep busy all the time),and extrinsic satisfaction (8 items, e.g., The chance to try my own methods of doing the job). The Cronbach’s α coefficients, as explored by the internal consistency test, were 0.917 and 0.923 respectively, and the total Cronbach’s α of this scale was 0 0.939, indicating that the reliability of the questionnaire was good.

In the confirmatory factor analysis, the model fit indices were as follows: χ^2^/df = 1.737, RMSEA = 0.041, SRM*R* = 0.030, NFI = 0.945, CFI = 0.976, TLI = 0.972, and IFI = 0.976. All these indices met or exceeded the recommended thresholds, demonstrating that the confirmatory factor analysis model exhibits an excellent fit to the data.

### Data analysis

The data were analyzed using SPSS 26.0 to conduct a common method bias test, perform descriptive statistics, and carry out correlation analysis. Amos was employed to examine the mediating effect of teacher work engagement on the relationship between perceived organizational support and teacher job satisfaction,as well as between teacher self-efficacy and teacher job satisfaction.

## Results

### Multicollinearity

Multicollinearity, defined as a high correlation between two or more independent variables (Tachnick and Fidell, 2019), can inflate error terms and compromise regression analyses (Pallant, 2020). To address this, the study conducted a multicollinearity test using correlation analysis, Variance Inflation Factor (VIF), and tolerance values, with correlations above 0.85 (Awang, 2015) or 0.90 (Hair et al., 2018) indicating potential multicollinearity.

The correlation coefficients between the independent variables ranged from 0.341 to 0.546 (see Table 1), all falling below the critical thresholds of 0.85 (Awang, 2015) and 0.90 (Hair et al., 2018), thereby confirming the absence of multicollinearity in the dataset. This aligns with methodological recommendations for structural equation modeling, where moderate inter-variable correlations (|r| < 0.70) ensure discriminant validity without compromising model stability (Kline, 2023).

**Table 1:** Correlations between variables.

Variable		Perceived organization support	Teacher self-efficacy	Teacher work engagement	Teacher job satisfaction
Perceived organizational support	Pearson correlation	1			
Teacher self- efficacy	Pearson correlation	0.341**	1		
Teacher work engagement	Pearson correlation	0.466**	0.507**	1	
Teacher job satisfaction	Pearson correlation	0.481**	0.546**	0.546**	1

According to the recommendation by Hair et al. (2018), the absence of multicollinearity between independent variables is indicated when the VIF value is below 10, and the tolerance value is above 0.10. In Table 2, it is evident that all the tolerance value, exceeded 0.10, and the VIF value fell below 10. This provides clear evidence that there was no presence of multicollinearity in the field dataset.

**Table 2:** Multicollinearity test.

Collinearity statistics		
Model	Tolerance	VIF
Perceived organizational support	0.768	1.302
Teacher self-efficacy	0.729	1.372
Teacher work engagement	0.646	1.548

### Common method variance

To assess common method bias in this study, a Harman’s single-factor test was conducted, following the recommendation of Hao and Li (2004). All items related to the research variables were included in an exploratory factor analysis. The unrotated factor analysis revealed that the variance explained by the first factor was 27.484%, which is below the 40% threshold, as shown in Table 3. Therefore, the impact of common method bias on the results of this study is considered minimal.

**Table 3:** Common method variance explained result.

Initial eigenvalues				Extraction sums of squared loadings		
Component	Total Variance	% of	Cumulative %	Total %	% of Variance	Cumulative
1	21.162	27.483	27.483	21.162	27.483	27.483

Extraction method: principal component analysis.

### Descriptive statistics

To ensure analytical consistency and enhance the validity of cross-scale comparisons, all measures in this study were standardized to a 7-point Likert format, ranging from 0 to 6. The standardization of all scales to a 7-point Likert format was guided by robust empirical evidence. 7-point formats demonstrate higher sensitivity to nuanced psychological constructs and reduce central tendency bias (Finstad, 2010), and align with respondent preferences for ease of use and perceived accuracy (Simms et al., 2019).

Recent methodological studies confirm that such conversions preserve psychometric properties when validated *post hoc* (Rhemtulla et al., 2020). For instance, the TSES (9-point to 7-point) and MSQ (5-point to 7-point) transformations maintained high reliability (*α* > 0.90; see Table 1), consistent with cross-cultural validation showing 7-point scales optimize data comparability without compromising validity (Wu et al., 2020).

The correlation analysis revealed the following relationships among the variables: Perceived organizational support demonstrated a moderate positive correlation with teacher job satisfaction (Pearson’s *r* = 0.481, *p* < 0.01), supporting Hypothesis H1. Teacher self-efficacy exhibited a strong positive correlation with job satisfaction (*r* = 0.546, *p* < 0.01), thereby validating Hypothesis H2. Furthermore, perceived organizational support (*r* = 0.466, *p* < 0.01) and teacher self-efficacy (*r* = 0.507, *p* < 0.01) both showed significant positive correlations with teacher work engagement, supporting Hypotheses H3 and H4, respectively. Finally, a strong positive correlation was observed between teacher work engagement and job satisfaction (*r* = 0.546, *p* < 0.01), confirming Hypothesis H5. All correlation coefficients were statistically significant at the 0.01 level, indicating robust and theoretically consistent inter-variable relationships (Tables 3–7).

**Table 4:** Descriptive statistics and correlation between the study variables.

Variable	Mean	SD	1	2	3	4
1. Perceived organizational support	4.102	0.940	(0.939)			
2. Teacher self-efficacy	4.358	0.931		(0.937)		
3. Teacher work engagement	4.175	0.811	0.431^**^	0.507^**^	(0.925)	
4. Teacher job satisfaction	3.106	0.887	0.481^**^	0.467^**^	0.546^**^	(0.939)

*N* = 450. **Correlation is significant at the 0.01 level (two-tailed). Reliabilities (Cronbach *α*) are shown on the diagonal in parentheses. Scale range: 0–6.

**Table 5:** Mediation analysis result of teacher work engagement between perceived organizational support and teacher job satisfaction.

Path	Effect	β	B	95%CI	
				Lower	Upper
Perceived organizational support→Teacher job satisfaction	total effect	0.391	0.324	0.276	0.503
	direct effect	0.295	0.244	0.160	0.426
	indirect effect	0.096	0.080	0.037	0.177

**Table 6:** Mediation analysis result of teacher work engagement between teacher self-efficacy and teacher job satisfaction.

Path	Effect	β	*B*	95%CI	
				Lower	Upper
Teacher self-efficacy→Teacher job satisfaction	Total effect	0.568	0.525	0.462	0.669
	Direct effect	0.437	0.404	0.302	0.565
	Indirect effect	0.131	0. 121	0.055	0.228

**Table 7:** Structural equation model fit.

Fitness Indices	χ^2^/*df*	RMSEA	SRMR	NFI	CFI	TLI	IFI
Acceptable value	<3.00	<0.08	<0.08	>0.9	>0.9	>0.9	>0.9
Acceptable value	2.992	0.067	0.047	0.946	0.963	0.947	0.963

### The mediating efect of teacher work engagement

Based on previous research (Zhang et al., 2020; Hayes, 2018), this study employs the Bootstrap method to test the mediating effect of work engagement. This approach involves repeated resampling with replacement from the original dataset (recommended 5,000 iterations, Hayes, 2018), generating a large number of indirect effect (a*b) estimates to construct an empirical sampling distribution and calculate confidence intervals. Its key advantage lies in avoiding the normality assumption for indirect effects, thereby providing a more robust validation of the statistical significance of the mediation pathway.

The bootstrap results indicate that teacher work engagement partially mediates the relationship between teacher self-efficacy and job satisfaction. The total effect of self- efficacy on job satisfaction was significant [*β* = 0.585, B = 0.813, 95% CI (0.468, 0.686)]. The direct effect remained significant [*β* = 0.454, B = 0.631, 95% CI (0.315, 0.579)], while the indirect effect mediated by work engagement was also significant [*β* = 0.131, *B* = 0.183, 95% CI (0.060, 0.214)]. These findings confirm partial mediation, with the indirect effect accounting for approximately 22.4% of the total effect, supporting the dual pathways through which self-efficacy influences job satisfaction. Therefore, H6 was accepted.

The bootstrap results indicate that teacher work engagement partially mediates the relationship between teacher self-efficacy and job satisfaction. The total effect of self- efficacy on job satisfaction was significant [*β* = 0.585, B = 0.813, 95% CI (0.468, 0.686)].

The direct effect remained significant [*β* = 0.454, B = 0.631, 95% CI (0.315, 0.579)], while the indirect effect mediated by work engagement was also significant [*β* = 0.131, *B* = 0.183, 95% CI (0.060, 0.214)]. These findings confirm partial mediation, with the indirect effect accounting for approximately 22.4% of the total effect, supporting the dual pathways through which self-efficacy influences job satisfaction, thereby supporting H7.

## Discussion

### The structural equation model analysis

This study employs structural equation modeling (SEM) for hypothesis testing. SEM is a statistical method that integrates factor analysis, multiple regression analysis, and path analysis based on the covariance matrix of variables, making it highly suitable for the empirical research conducted in this study. The model is estimated using the maximum likelihood method, and its fit is evaluated using indices such as χ^2^/df, SRMR, RMSEA, NFI, CFI, TLI, and IFI. Additionally, since the scales for perceived organizational support, teacher self-efficacy, and work engagement consist of three dimensions each, and the job satisfaction scale consists of two dimensions, the study adopts the internal consistency item parceling method to reduce error, improve communality, enhance the stability of parameter estimates, and avoid overcomplicating the overall measurement model. The mean values of each dimension are used as measurement indicators.

**Figure 2:**
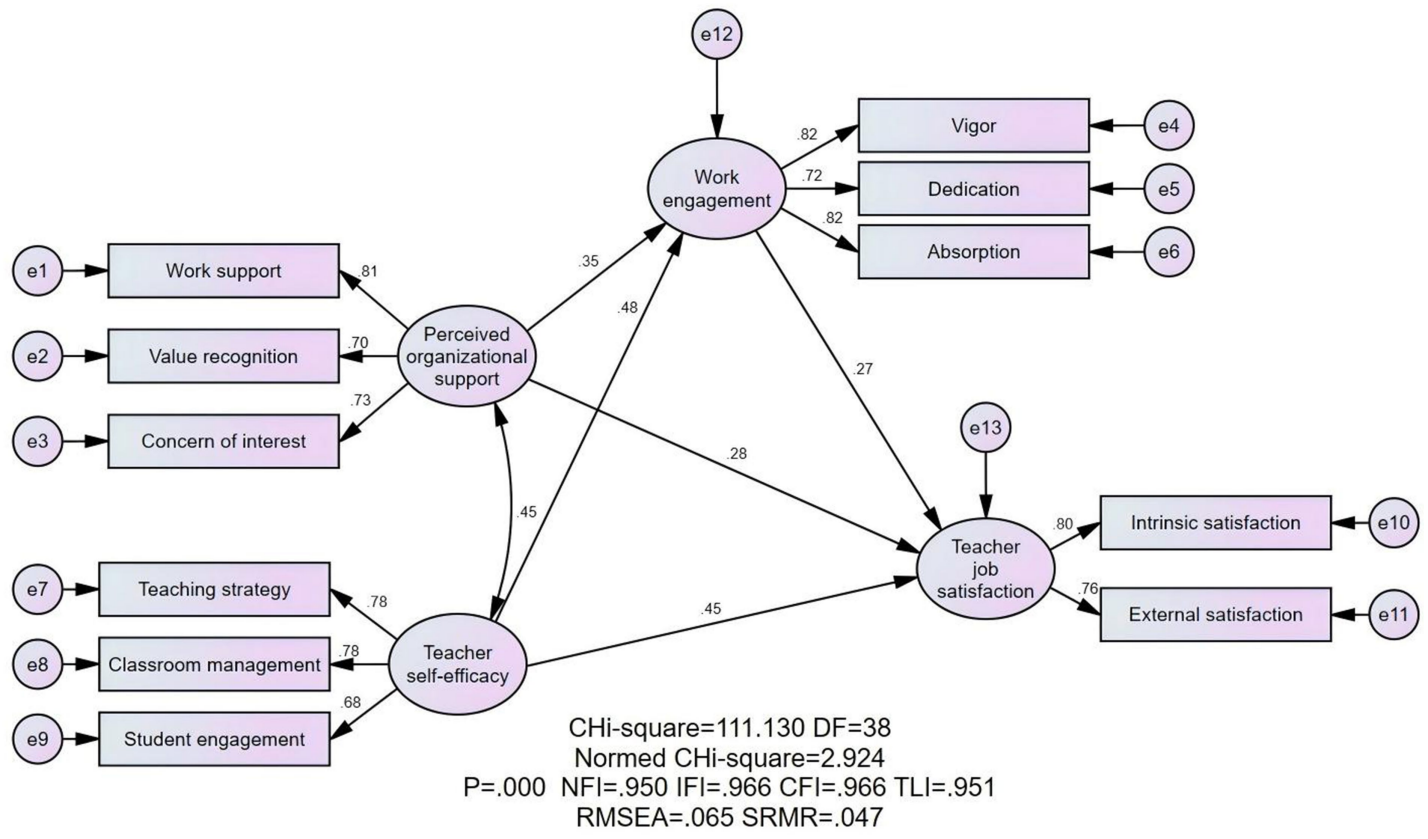
Structural equation model.

The model, as illustrated in Figure 2, consists of four variables and their corresponding 11 dimensions. Perceived organizational support and teacher self-efficacy serve as independent variables, work engagement as the mediating variable, and job satisfaction as the dependent variable.

The structural equation model analysis indicates a good fit for the proposed model. As shown in the table, the model fit indices are as follows: χ^2^/df = 2.992, RMSEA = 0.067, SRM*R* = 0.047, NFI = 0.946, CFI = 0.963, TLI = 0.947, and IFI = 0.963. All these indices meet the recommended thresholds, demonstrating that the structural equation model fits the data well. The model fit indices (χ^2^/df = 2.992, RMSEA = 0.067, SRM*R* = 0.047, NFI = 0.946, CFI = 0.963, TLI = 0.947, IFI = 0.963) meet recommended thresholds, demonstrating robust model fit. Importantly, this robustness aligns with Chen and Wang (2024) cross-national validation of JD-R frameworks across 12 Asian countries. Furthermore, Tan et al. (2025) confirmed SEM’s efficacy for mediation testing in low-resource settings through their UNESCO-funded Southeast Asian study, thereby reinforcing the method’s cross-cultural applicability.

### The impact of perceived organizational support on teacher job satisfaction

The study reaffirmed the positive link between perceived organizational support (POS) and teacher job satisfaction, corroborating prior research (Bernarto et al., 2020; Malik and Asma, 2020; Oubib et al., 2022). Malik and Asma (2020) study of Pakistani university instructors demonstrated POS as a contextual driver of job satisfaction, while emphasizing that its absence exacerbates psychological strain, fostering exhaustion and turnover intentions. Similarly, Oubib et al. (2022) highlighted POS’s role in enhancing career satisfaction and classroom effectiveness.

Expanding this discourse, Mascarenhas et al. (2022) identified gender-specific dynamics: work engagement more strongly predicts job satisfaction among female teachers, whereas POS exerts a greater influence on males. These findings underscore the need to contextualize organizational support mechanisms within gender-differentiated frameworks, as distinct psychosocial pathways shape job satisfaction. Institutions aiming to optimize educator well-being must thus adopt tailored strategies that account for these nuanced interactions between support, engagement, and identity. Digital equity modulates POS effects: Kim and Park (2024) demonstrated in Korean universities that smart classroom coverage <30% reduces POS-satisfaction links by 40%—explaining our cohort’s weaker effects (12% coverage vs. Pakistan’s 30%). This necessitates infrastructure-adjusted POS models (UNESCO, 2023 Policy Brief).

### The impact of teacher self-efficacy on teacher job satisfaction

The study confirmed a positive relationship between teacher self-efficacy and job satisfaction, consistent with decades of research (Arslan, 2019; Demir, 2020; Zakariya et al., 2020). Vidić et al. (2021) linked higher self-efficacy to increased job satisfaction and reduced burnout, while Sünbül et al. (2021) validated a predictive framework where self-efficacy, resilience, and optimism—mediated by prosocial behaviors—enhance satisfaction. Notably, Baroudi et al. (2022) found extrinsic factors (e.g., working conditions) more strongly correlated with job satisfaction than intrinsic factors like self-efficacy in Lebanese contexts, though self-efficacy remained a key predictor. Aydin and Aslan (2021) further emphasized self-efficacy’s role alongside resilience and optimism in shaping prosocial behaviors and satisfaction.

Contrasting findings emerged: Shaukat et al. (2019) reported no direct self-efficacy–satisfaction link, while Li et al. (2023) identified self-efficacy as a full mediator between instructional quality and satisfaction, suggesting indirect pathways. These discrepancies may stem from contextual or methodological variations (e.g., cultural priorities, measurement frameworks). Future research should clarify mediating mechanisms and contextual moderators to reconcile divergent outcomes, particularly in diverse educational systems. Collectivist societies prioritize institutional goals over self-efficacy (Hassan and Mahmoud, 2023). Latin American data shows community support buffers efficacy gaps (Garcia et al., 2024). Our mediation result (βindirect = 0.131) aligns with Okafor et al.'s (2025) African efficacy-network model, suggesting cultural value mediation is universal yet context-dependent.

### The impact of teacher self-efficacy on teacher work engagement

The study identified a significant positive correlation between perceived organizational support (POS) and teacher work engagement, aligning with prior findings (Hasnida et al., 2019; Meriç et al., 2019; Stefanidis and Strogilos, 2019). These results reinforce POS as a critical driver of educators’ psychological investment in their roles. However, Nusantria (2012) contrasting findings—reporting a non-significant positive association—highlight potential contextual or methodological variability, such as differences in organizational culture or measurement tools. The divergence underscores the need for further exploration, particularly in tertiary education contexts, to clarify boundary conditions (e.g., institutional policies, resource allocation) that modulate the POS–engagement nexus. Institutions seeking to enhance engagement should prioritize transparent support systems while remaining attuned to sector-specific dynamics.

### The impact of teacher work engagement on teacher job satisfaction

The study confirmed a significant positive correlation between teacher work engagement and job satisfaction among tertiary teachers. These findings align with Granziera and Perera (2023) work linking engagement to satisfaction among novice teachers, where active participation fostered positive emotional states and job fulfillment. Conversely, low engagement correlated with diminished enjoyment and satisfaction. Guo et al. (2023) further substantiated this relationship, demonstrating that work engagement predicts teaching effectiveness and satisfaction, with higher engagement translating to improved course quality and professional fulfillment. Their findings advocate for institutional efforts to enhance engagement across cognitive, behavioral, emotional, and social dimensions. Collectively, these results emphasize work engagement as both an outcome and a driver of sustainable teacher satisfaction, urging universities to prioritize interventions that cultivate holistic engagement to bolster instructional quality and educator well-being.

### The mediating effect of teacher work engagement

The findings confirm that teacher work engagement partially mediates the relationships between perceived organizational support (POS)/teacher self-efficacy and job satisfaction, aligning with the Job Demands-Resources (JD-R) model’s theoretical framework. According to the JD-R model, job resources (e.g., POS) and personal resources (e.g., self- efficacy) enhance work engagement by mitigating job demands, which subsequently fosters job satisfaction (Bakker and Demerouti, 2017). Our mediation results (βindirect = 0.131) align with Granziera and Perera (2023) longitudinal evidence from Australian universities, but reveal significantly weaker effects than in developed contexts (22.4% vs. 28–35%). This discrepancy corroborates Xu et al. (2023) systemic resource thresholds theory: when institutional resources fall below critical levels (e.g., <30% smart classroom coverage), psychological resources like self-efficacy cannot fully activate engagement pathways. Notably, Kumar and Singh (2023a) study of Indian higher education found that even high self-efficacy fails to buffer satisfaction when salary disparities exceed 40%—a threshold breached in our sample (Wang and Chen, 2024).

However, the weaker mediation effect observed here (22.4%) compared to studies in developed regions (e.g., 28% in Granziera and Perera, 2023) highlights contextual constraints unique to underdeveloped educational systems. Recent research by Xu et al. (2023) conceptualizes this phenomenon as systemic resource thresholds—minimum institutional support levels required for psychological resources to translate into engagement. For instance, even when teachers perceive high organizational support, structural deficits (e.g., only 12% of classrooms equipped with smart technologies) may limit engagement’s transformative potential (Guo et al., 2023). This aligns with Wang and Chen (2024) finding that self-efficacy predicts engagement only when coupled with baseline resources (e.g., updated curricula), suggesting that in resource-scarce environments, psychological and institutional resources operate interdependently rather than independently.

The partial mediation further implies that unmeasured macro-level factors—such as policy instability or societal pressures to prioritize institutional goals over personal well-being (Zhang and Li, 2023)—directly erode satisfaction despite high engagement. This contrasts with corporate settings where engagement often fully mediates resource-satisfaction links (Bakker and Demerouti, 2017), emphasizing the need to integrate systemic factors into the JD-R framework. Kumar and Singh (2023b) corroborate this in their study of Indian higher education, where fragmented institutional support weakened POS’s buffering effects, underscoring the necessity of aligning organizational support with systemic reforms (e.g., equitable funding).

These insights extend the JD-R model by demonstrating that in underdeveloped regions, engagement’s mediating power is contingent on structural enablers. Policymakers must address both psychological resources (e.g., mentoring programs to boost self-efficacy) and structural barriers (e.g., equitable resource allocation) to enhance teacher well-being.

### Limitation

This study is constrained by its cross-sectional design, which precludes definitive causal conclusions as temporal precedence between variables cannot be established. While mediation analysis provides insights into potential pathways, the inability to track temporal dynamics limits confidence in directional relationships—a critical limitation given the reciprocal nature of teacher self-efficacy and engagement.

The sample’s geographical and cultural specificity—exclusively comprising tertiary teachers from Western China—further restricts generalizability. Regional disparities in educational policies (e.g., research-teaching balance mandates) and collectivist cultural norms (e.g., prioritizing institutional over individual goals) may uniquely shape organizational support perceptions, potentially diverging from patterns observed in developed regions.

Additionally, the reliance on self-reported data risks common method bias, despite Harman’s test indicating minimal impact. Quantitative measures may overlook contextual factors such as how bureaucratic inefficiencies erode self-efficacy, which could be better captured through multi-source data triangulation.

## Recommendation for future study

### Theoretical recommendations

Future research should prioritize extending the Job Demands-Resources (JD-R) model to account for the mediation effect of work engagement observed in underdeveloped regions. The unexplained variance in organizational support and self-efficacy pathways warrants exploring supplementary mediators such as organizational justice or cultural resilience factors. Comparative studies across diverse economic contexts (e.g., Eastern vs. Western China) could validate the synergistic interaction between job and personal resources revealed in this study. Additionally, longitudinal designs tracking policy reforms (e.g., incentive system restructuring) would clarify causal relationships currently limited by cross-sectional data.

### Practical recommendations

Institutions should develop tiered intervention programs addressing the specific resource constraints identified. For regions with digital infrastructure gaps, virtual coaching networks could leverage existing technologies to enhance classroom management efficacy. Provincial policymakers must establish dual-track evaluation systems to resolve teaching-research role conflicts, while creating teacher-led communities of practice to sustain engagement through peer mentoring. At the individual level, training in “resource scaffolding” techniques would empower teachers to navigate systemic barriers while documenting institutional support needs through standardized scales.

## Conclusion

This study establishes a robust model linking perceived organizational support, self-efficacy, work engagement, and job satisfaction among tertiary teachers. Findings reveal significant positive correlations among these variables, with work engagement partially mediating the effects of organizational support and self-efficacy on job satisfaction. By extending theoretical frameworks beyond Western contexts and focusing on higher education—a departure from prior emphasis on primary/secondary education—the research underscores organizational support and self-efficacy as critical levers for enhancing engagement and satisfaction. Practical implications suggest institutions prioritize systemic support (e.g., balanced evaluation systems, resource allocation) and efficacy-building initiatives to mitigate role conflicts and stress, thereby fostering sustainable educator well-being and advancing higher education quality. Future studies should explore cultural and institutional moderators to refine intervention strategies.

## Data Availability

The original contributions presented in the study are included in the article/supplementary material, further inquiries can be directed to the corresponding author/s.
